# A Systematic Study on Pretraining Strategies for Low-Label Remote Sensing Image Semantic Segmentation

**DOI:** 10.3390/s26041385

**Published:** 2026-02-22

**Authors:** Peizhuo Liu, Hongbo Zhu, Xiaofei Mi, Jian Yang, Yuke Meng, Huijie Zhao, Xingfa Gu

**Affiliations:** 1 School of Instrumentation and Optoelectronic Engineering, Beihang University, Beijing 100191, China; liupeizhuo@buaa.edu.cn; 2 Aerospace Information Research Institute, Chinese Academy of Sciences, Beijing 100094, China; zhuhb@aircas.ac.cn (H.Z.); mixf@aircas.ac.cn (X.M.); yangjian@aircas.ac.cn (J.Y.); 3 State Key Laboratory of Information Engineering in Surveying, Mapping and Remote Sensing, Wuhan University, Wuhan 430072, China; mengyuke@whu.edu.cn; 4 School of Artificial Intelligence, Beihang University, Beijing 100191, China; hjzhao@buaa.edu.cn; 5 School of Geography and Remote Sensing, Guangzhou University, Guangzhou 510006, China

**Keywords:** remote sensing, semantic segmentation, limited labels, self-supervised learning, two-stage pretraining, swin transformer

## Abstract

This paper addresses the critical challenge of semantic segmentation for remote sensing images (RSIs) under extremely limited labeled data. A high-quality initial model is paramount for downstream semi-supervised or weakly supervised learning paradigms, as it mitigates error propagation from the outset. We conducted a systematic investigation into self-supervised pretraining to serve this precise need. Within the low-label regime, we identify and tackle two pivotal factors limiting performance: (1) the domain shift between large-scale pretraining data and specific target tasks, and (2) the deficiency in local feature learning caused by large-window masking in visual foundation model (VFM) pretraining. To resolve these issues, we first benchmark various pretraining strategies, demonstrating that a two-phase General-Purpose Pretraining (GPPT) followed by Domain-Adaptive Pretraining (DAPT) framework is optimal, significantly outperforming both single-phase methods and the existing two-phase paradigm initialized from ImageNet. Subsequently, we propose an Edge-Guided Masked Image Modeling (EGMIM) method for the DAPT phase, which explicitly integrates edge priors to guide the masking and reconstruction process, thereby enhancing the model’s capability to capture fine-grained local structures. Extensive experiments on four RSI benchmarks validate the effectiveness of our approach, showing consistent and substantial gains, particularly in extreme low-label scenarios. Beyond empirical results, we provide in-depth mechanistic analyses to explain the synergistic roles of GPPT and DAPT.

## 1. Introduction

Semantic segmentation of high-resolution remote sensing images (RSIs) is crucial for applications such as smart cities, environmental monitoring, precision agriculture, and disaster assessment. In recent years, deep learning methods, particularly convolutional neural networks (CNNs), have become the mainstream approach for this task. As research progresses, transformer-based Visual Foundation Models (VFMs) [1,2] have demonstrated significant potential in RSI segmentation due to their powerful global modeling capability. However, VFMs typically contain a vast number of parameters, requiring massive amounts of annotated data for training. Acquiring pixel-level annotations for RSIs is extremely time-consuming and labor-intensive, posing a fundamental barrier to deploying advanced models.

To reduce reliance on expensive annotations, various strategies have been proposed. Among them, semi-supervised learning [3] and weakly supervised learning [4] are two primary pathways. Their training processes both involve using the available limited labels (or sparse labels derived from weak supervision) to train an initial teacher network, which is subsequently used to generate pseudo-labels for expanding the training set. Therefore, the quality of this initial teacher network is critical. However, when trained solely on very few labels, such an initial network is inherently under-trained and often performs poorly, which directly compromises the quality of the pseudo-labels it generates. This creates a foundational bottleneck that limits the entire learning framework. Yet, most existing works focus on improving the algorithms for generating or utilizing pseudo-labels, often overlooking a more fundamental opportunity: how to obtain a better-performing initial teacher model before the pseudo-labeling cycle begins.

This paper explores a complementary yet distinct path. Instead of focusing on the pseudo-label generation or utilization mechanisms within semi-/weakly supervised frameworks, we shift focus upstream to the stage before any pseudo-label is produced—the pretraining stage. We argue that the core issue of an underperforming initial model lies in its insufficient intrinsic representational capacity when learned from scratch with minimal labels. By enhancing the efficacy of Self-Supervised Learning (SSL) [5] specifically under low-label conditions, we can obtain a substantially more powerful initial teacher network. Such an enhanced model can serve as a superior starting point, effectively empowering subsequent semi-supervised or weakly supervised learning pipelines by providing higher-quality pseudo-labels from the very beginning.

Therefore, this work aims to explore an effective self-supervised pretraining method tailored for low-label scenarios. Positioned as such, we identify two critical issues affecting pretrained model performance in this setting.

First, the domain shift inherent in transferring pretrained features to a specific target task is particularly detrimental under limited labeled data. Current pretraining strategies can be conceptually divided into single-phase and two-phase frameworks. Single-phase frameworks, whether initialized from models pretrained on natural images (e.g., ImageNet [6]) or on large-scale RSI collections, often struggle with the distribution discrepancy when finetuned directly on a target domain. While the two-phase framework, which introduces an intermediate domain-adaptive pretraining (DAPT) [7,8,9] phase on unlabeled target data, has been explored to bridge this gap, existing instantiations of this framework (e.g., initializing from ImageNet pretrained weights) are suboptimal as they start from a foundation not inherently tailored to the remote sensing domain. This highlights a critical gap: the lack of a systematic investigation into a two-phase framework that is principally designed for RSI from the ground up—i.e., one that begins with a general-purpose pretraining (GPPT) phase on a massive and diverse RSI corpus to learn domain-native representations, followed by a dedicated DAPT phase for target specialization.

**Figure 1:**
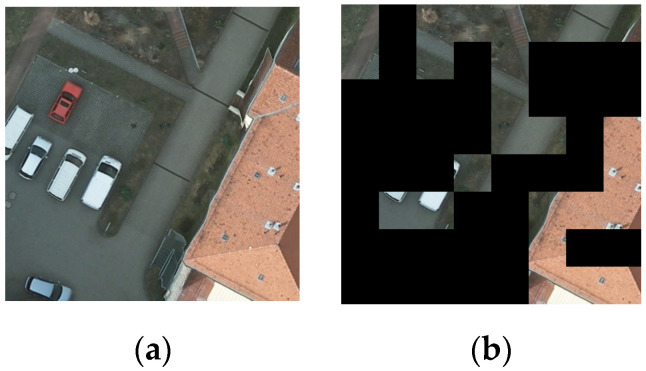
Limitations of large-window masking in high-resolution RSI. (**a**) Original image; (**b**) standard SimMIM [11] mask. The red vehicle visible in (**a**) is completely occluded in (**b**), demonstrating how large window masking strategy impedes local feature learning.

Second, VFMs typically employ Masked Image Modeling (MIM) [10,11] as their pretraining objective. While empirical studies show that large masking windows generally yield optimal performance for natural images—where objects often occupy substantial regions and can preserve most visual information after masking—this strategy is misaligned with the requirements of semantic segmentation for high-resolution RSIs. RSIs frequently contain numerous small objects and fine local structures, which are often completely obscured by large masking windows, as exemplified by the red vehicle in Figure 1b. This prevents the model from learning robust feature representations for these critical, localized elements. We contend that this represents a fundamental misalignment: the standard MIM objective, with its unstructured random masking, does not incentivize the model to learn the fine-grained, structurally aware features that are paramount for dense prediction tasks like RSI semantic segmentation. This limitation becomes particularly detrimental in low-label scenarios, where the quality of the pretrained features directly dictate the performance ceiling after finetuning.

**Figure 2:**
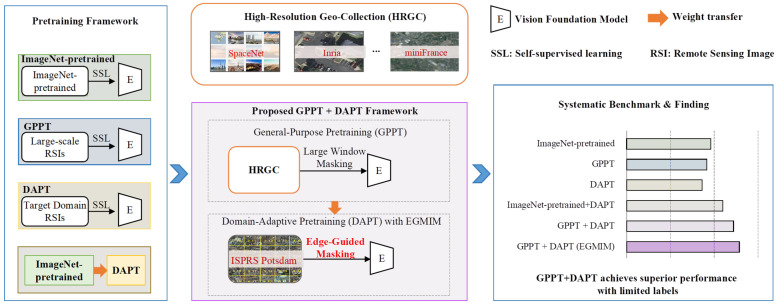
Conceptual diagram of this study. We systematically benchmark various pretraining strategies (**left**) and demonstrate that the proposed GPPT+DAPT framework with EGMIM (**middle**) yields superior segmentation performance under limited labeled data, as validated by experimental benchmarks and analysis (**right**).

To address these challenges and rigorously evaluate the pretraining strategies, this paper conducts a comprehensive empirical study. Rather than solely proposing another novel framework, we systematically benchmark five representative pretraining configurations, including single-phase approaches and the two-phase paradigms of ImageNet+DAPT and our proposed GPPT+DAPT. This comparative approach allows us to unequivocally identify the optimal strategy and, more importantly, to uncover the underlying mechanisms through in-depth analysis of attention patterns, feature distributions and parameter difference analysis. Furthermore, we propose an Edge-Guided Masked Image Modeling (EGMIM) method in the DAPT phase. It utilizes low-level edge information as prior to guide the masking process, compelling the model to reconstruct and learn critical local image features. This further improves segmentation performance under low-annotation scenarios. An overview of the proposed pretraining framework and the comparative study is presented in Figure 2.

The main contributions of this work are threefold:

(1) We establish that the full two-phase framework, GPPT followed by DAPT, represents the optimal pretraining strategy. Through a comprehensive benchmark, we demonstrate that this approach significantly outperforms not only single-phase methods but also the previously explored two-phase paradigm initialized from ImageNet, particularly in low-label scenarios.

(2) We propose EGMIM—a novel pretraining objective for the DAPT phase. By integrating edge prior information into the masking strategy, it significantly enhances the learning of local feature representations, leading to improved segmentation performance in low-annotation settings.

(3) We provide mechanistic insights into the two-phase pretraining process. From the perspectives of attention patterns, feature distribution shifts, and model parameter changes, we dissect how GPPT and DAPT collaboratively enhance feature representations, explaining the superiority of the complete framework.

## 2. Related Works

### 2.1. Research Directions for Reducing Annotation Cost

Research efforts to alleviate the dependency on pixel-level annotations for RSI semantic segmentation primarily follow three distinct pathways [12], which are crucial to differentiate from the approach taken in this work.

Semi-supervised semantic segmentation typically employs self-training [13] or consistency regularization [14] frameworks, where a teacher model generates pseudo-labels for unlabeled data. A fundamental limitation persists: the initial teacher model, trained solely on a very small labeled set, carries inherent inaccuracies. These errors are propagated into the pseudo-labels from the outset, hampering the entire training process.

Weakly supervised semantic segmentation seeks to replace pixel-level annotations with cheaper, weaker forms of supervision (e.g., scribbles [15], image-level tags [16]). These weak labels are often converted into sparse labels via existing techniques [17] to train an initial teacher network, which is then refined through self-training. Consequently, its performance is also bottlenecked by this suboptimal initial model.

Unsupervised Domain Adaptation (UDA) for semantic segmentation [18] aims to transfer a model learned from a labeled source domain to an unlabeled target domain by minimizing the distribution gap between them. Predominant methods often introduce adversarial training at the feature level to learn domain-invariant representations [19]. Although the adversarial loss itself is unsupervised, the UDA paradigm fundamentally relies on the availability of a labeled source domain.

A critical observation is that both semi-supervised and weakly supervised methods focus their innovation on the downstream training framework. However, they often overlook a root cause of poor performance in low-data regimes: the lack of optimization for the initial model’s representation power. An underperforming initial model generates low quality pseudo-labels, creating a flawed starting point that compromises subsequent learning. This work explores a complementary yet distinct path. We explore how self-supervised pretraining—a paradigm typically ignored by the aforementioned frameworks—can directly and significantly enhance the initial model’s performance before any downstream training begins. Therefore, our proposed pretraining method serves as a powerful front-end enhancement, solidifying the starting point for any subsequent semi-supervised or weakly supervised pipeline.

### 2.2. Pretraining Algorithms for RSI Segmentation

Contrastive learning (CL) and image reconstruction are two main stream methods for self-supervised pretraining [5]. Several studies have sought to introduce localized CL methods to adapt to semantic segmentation tasks [20,21,22,23]. For example, CMID [20] proposed a local matching of positive sample pairs to enhance the BYOL [24] method. However, these methods face challenges: they are often designed for smaller CNNs, their performance is highly sensitive to augmentations (which may harm RSI spectral integrity), and defining the optimal granularity for local contrast is non-trivial. Therefore, this work focuses on pixel-level reconstruction paradigms, particularly MIM, which is better suited for capturing the fine-grained details necessary for RSI segmentation.

Early techniques use classical auto-encoder [25,26] or image inpainting [27,28] to enhance the performance of CNNs. Mask Image Modeling is a new branch suitable for VFMs. In Scale-MAE [29], the ViT [1] was pretrained by a variant of MAE [10], which incorporated the resolution scale of RSIs into position encoding and reconstructed the masks of the original image with a multiscale decoder. SatMAE [30] integrated a novel spectral encoding with positional encoding in MAE, thus enabling the processing of multispectral images with varying resolutions across different spectral bands. SatMAE++ [31] modified MAE to reconstruct images at multiple scales simultaneously. ViTAEv2 [32] and BFM [33] investigated the performance improvement achieved by changing the network structure of the ViT and increasing its width and depth, respectively. FG-MAE [34] proposed reconstructing image features such as HOG instead of the original image to avoid the influence of speckle noise in SAR images when using MAE. RingMo [35] pretrained the swin transformer [2] with SimMIM [11] and verified its applicability in several remote sensing interpretation tasks. CxtMIM [36] added a context consistency constraint to SimMIM to provide additional context information.

Several studies constrained the pretraining process in a multitask learning paradigm. For example, GeRSP [37] integrated both CL with RSIs and supervised learning with ImageNet [6]. Some studies [38,39,40] pretrained models by optimizing both image reconstruction and data distillation [24,41]. Cross-Scale MAE [42] added cross-scale consistency constraints besides image reconstruction. These multitask paradigms often require more computational resources and are sensitive to loss weighting.

While the MIM-based pretraining methods mentioned above focus on performance under fully annotated data, they lack designs specifically tailored for low-label scenarios, and their performance under such conditions has not been systematically compared. Moreover, a key limitation of existing MIM methods is their reliance on a standard large-window masking strategy. We find that this design causes many local detail features in high-resolution RSIs to remain unlearned. This issue is subtle when abundant labels are available, but significantly damages the model’s performance under limited labeled data. Our study addresses this critical gap by introducing edge-guided prior information into the masking strategy.

### 2.3. Pretraining Frameworks for RSI

**Figure 3:**
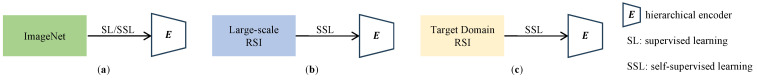
Single-phase pretraining frameworks. (**a**) ImageNet-pretrained; (**b**) GPPT on large-scale RSIs; (**c**) DAPT on target domain RSIs.

Existing pretraining frameworks can be categorized by the number of pretraining phases and the data domain utilized. Single-phase strategies, which involve just one phase of SSL followed by supervised finetuning, are prevalent. These include: initializing with features learned from natural images, such as ImageNet (Figure 3a); leveraging large-scale RSIs for GPPT, which builds a domain-specific foundation (Figure 3b) [35,43,44]; or performing DAPT directly on the target domain data itself (Figure 3c) [20,21]. While straightforward, these single-phase approaches face inherent limitations: strategies based on external datasets (ImageNet or general RSIs) suffer from domain shift when applied to a specific target, while target-specific DAPT risks overfitting and lacks generalization due to limited data.

Recognizing these limitations, the two-phase paradigm has emerged as a more powerful alternative. This paradigm strategically combines the phases above, first establishing a robust feature foundation and then specializing it for the target domain. Prior works [7,8,9] have explored a pipeline that sequentially combines ImageNet-pretraining (as in Figure 3a) with a subsequent DAPT phase. However, these studies primarily present it as an alternative strategy, lacking a systematic comparison against a principled, RSI-native baseline—specifically, the framework of GPPT followed by DAPT—and crucially, an in-depth analysis of the underlying mechanisms.

Our work addresses this gap. We systematically benchmark the two-phase paradigm, demonstrating that initializing it with an RSI-native GPPT foundation (i.e., the integrated GPPT+DAPT framework) yields superior performance. Beyond empirical comparison, we provide a mechanistic examination of how GPPT and DAPT collaboratively reshape feature representations, offering a deeper understanding of why this specific framework is optimal.

It is crucial to differentiate the DAPT phase discussed in this work from UDA. While both involve unlabeled target domain data, they address different problems and are applied at different stages. UDA for semantic segmentation typically involves a model trained on a labeled source domain and is adapted to an unlabeled target domain, often requiring complex adversarial training or self-training mechanisms. In contrast, the DAPT phase in our work is a self-supervised representation learning step applied to a generically pretrained backbone using only unlabeled target domain data, before any task-specific finetuning. This distinction highlights that our focus is on adaptive pretraining at the representation level, rather than domain adaptation of a task-specific model.

## 3. Methods

### 3.1. Overview

The proposed approach employs a standard segmentation architecture comprising a hierarchical backbone network for multi-scale feature extraction and a segmentation head for semantic decoding. As the backbone network fundamentally governs the upper performance bound of the model, we adopted swinV2-B [45] from several prevailing VFMs. This choice was motivated by its hierarchical encoder design, which inherently generates multi-scale feature representations while resolving the training instability limitations of the original swin transformer. The input image is partitioned into non-overlapping 4 × 4 patches, each linearly projected into 128-dimensional feature vectors through a patch embedding layer. The serialized image features are then processed through four hierarchical stages, each containing swinV2 transformer layers with [4,8,16,32] attention heads, respectively. In each swinV2 layer, the attention scores for each head in the multi-head self-attention module is computed as:

(1) $$attention\;{score}_{i}\left(K_{i},Q_{i}\right)=Softmax\left(\frac{Q_{i}K_{i}^{T}}{\sqrt{d}}+B_{i}\right),\;i=1,2,\ldots ,h$$

where h is the number of heads in the self-attention module, $B_{i}\in R^{M^{2}\times M^{2}}$ is the relative position bias (RPB), $Q_{i},K_{i}\in R^{M^{2}\times d}$ are the query and key matrices, *d* is the query dimension, and *M* is the default shift window size set to 12. The swinV2-B architecture contains approximately 88 million parameters.

**Figure 4:**
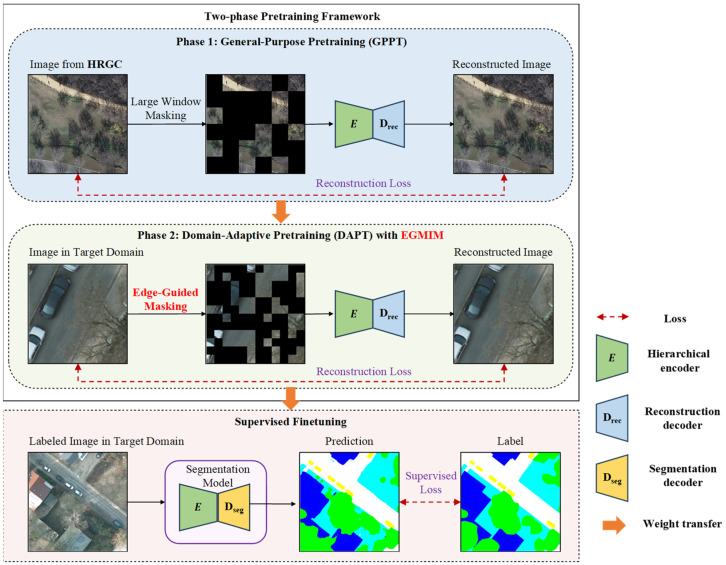
Framework illustrating the transition from general-purpose to domain-adaptive pretraining, culminating in task-specific finetuning for RSI segmentation.

As illustrated in Figure 4, we propose and employ a dedicated two-phase pretraining framework designed to enhance segmentation performance with limited annotations. This framework sequentially combines GPPT on large-scale RSIs with a DAPT phase on the unlabeled target data, followed by supervised finetuning on the available labeled data. The GPPT phase establishes a domain-relevant foundational model. The subsequent DAPT phase incorporates our proposed EGMIM method, which is designed to overcome the limitation of standard large-window masking in capturing fine-grained local features. EGMIM guides the reconstruction towards edge-rich, structurally salient regions. This cohesive framework is designed to collectively mitigate domain shift and improve local feature representation, thereby achieving higher segmentation accuracy after supervised finetuning, especially in low-label scenarios.

**Figure 5:**
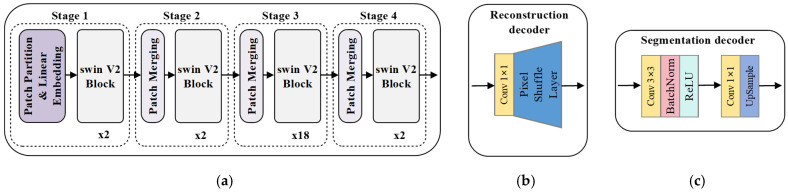
Schematic of the key network components: (**a**) SwinV2-B backbone, (**b**) reconstruction decoder, and (**c**) segmentation decoder.

The detailed network architectures are depicted in Figure 5. The reconstruction decoder adopts a lightweight design, comprising a convolutional layer followed by a pixel shuffle layer [46]. For the supervised finetuning, we employ a nonlinear fully convolutional network (FCN) [47] with two convolutional layers as the segmentation decoder. The input to the segmentation decoder was selected from the third-stage output (corresponding to the 22nd transformer layer) of the SwinV2-B backbone. This choice was driven by its optimal trade-off between feature map resolution and channel depth: the third stage preserves larger spatial dimensions compared to the fourth stage, while avoiding the doubled channel count of the fourth stage, which would increase the parameters of the segmentation decoder and compromise performance in low-label scenarios.

### 3.2. General-Purpose Pretraining

To explore feature representation for high-resolution RSIs, we constructed the High-Resolution Geo-Collection (HRGC) by aggregating diverse publicly available remote sensing datasets (see Table 1). HRGC primarily contains imagery with resolutions between 0.1 and 0.5 m from various platforms, focusing on common urban and rural landscapes to establish a general-purpose visual corpus. It is important to note that as an aggregation of existing benchmarks, HRGC inherits their limitations, including a lack of precise temporal metadata (e.g., exact season or year) and variations in original radiometric and geometric processing levels.

During compilation, we made practical curation decisions to prioritize learning robust features. Specifically, we excluded cities in SpaceNet with highly specific or information-scarce scenes that were present in some source datasets, such as Moscow (due to extensive snow cover obscuring ground objects) and Khartoum (due to its distinctive, homogenous arid landscape). This was done to avoid potential bias stemming from overly specialized visual characteristics.

All HRGC images were processed into 192 × 192 pixel slices with a 160-pixel stride, generating approximately 2.3 million samples. This extensive collection enables the swinV2 backbone to learn robust, generalizable features for high-resolution analysis while maintaining full reproducibility through publicly accessible data sources. To mitigate substantial radiometric disparities across sub-datasets while preserving their original spectral distributions, our primary preprocessing step was to normalize all pixel values uniformly to the [−1, +1] range. We deliberately did not perform unified radiometric or geometric corrections, as each constituent dataset was already of sufficient quality for its original purpose. We posit that learning from data with natural, low-level heterogeneity may even enhance the robustness of the feature extractor.

The GPPT phase employs the SimMIM method with two empirically determined parameters: mask window size $W_{p}$ default to 32 and masking ratio *R* default to 0.6. This method uniformly cuts input images into non-overlapping windows of size $W_{p}$ and randomly masks these windows with ratio *R* (see Figure 1b). The masked image is then encoded by the SwinV2-B backbone. A reconstruction decoder subsequently produces a recovered image. The optimization objective is to minimize the L1 loss between the original and the reconstructed pixels within the masked regions.

### 3.3. Domain-Adaptive Pretraining

In real-world applications, target domain RSIs are typically captured by a single sensor platform and constitute substantially smaller datasets than those used in the GPPT phase. To mitigate the inherent domain shift between the GPPT data and target domain imagery, we incorporate a DAPT phase as the second phase of our pretraining framework. The concept of applying second-phase, target-domain-specific self-supervised pretraining was explored to bridge domain gaps [7,8,9], which adopted ImageNet pretraining followed by DAPT. Our work distinguishes itself by applying DAPT after a strong, domain-native GPPT foundation and enhancing it with our novel EGMIM objective, forming the integrated GPPT+DAPT framework under systematic benchmarking.

DAPT is implemented in a simple yet effective manner in our work: the backbone network and reconstruction decoder pretrained during GPPT are used as initialization and further pretrained on unlabeled target domain data. Conceptually, DAPT can be viewed as unsupervised finetuning on target domain data. This approach preserves the general feature representations learned during GPPT while adapting them to the specific characteristics of the target domain. The term “domain-adaptive” refers to this feature-level adaptation of the backbone network. A key distinction between DAPT and the feature-level adaptation commonly used in UDA methods lies in the training objective and stage. While UDA for segmentation typically adapts a task-specific model (trained on a labeled source domain) to an unlabeled target domain, often using adversarial alignment losses, our DAPT is a pure self-supervised representation learning step applied to a generically pretrained backbone using only unlabeled target data, before any task-specific fine-tuning begins.

For a specific segmentation task, we enhance the GPPT-pretrained backbone by performing DAPT on its unlabeled images before supervised finetuning. Owing to the comprehensive training in the GPPT phase, DAPT typically converges rapidly with relatively few iterations, requiring substantially less computational time (see Section 4.6).

Although conventional SimMIM can be applied in DAPT, we propose EGMIM (detailed in Section 3.4) to further enhance segmentation performance in low-data regimes by guiding the model to learn more transferable local features.

### 3.4. EGMIM

The limitation of large-window masking, as illustrated in Figure 1, stems from its failure to encourage the learning of fine-grained features. Standard MIM treats all image regions equally, which is suboptimal for cultivating the discriminative representations required for RSI understanding. To better align the self-supervised pretraining objective with the needs of downstream tasks, we propose the EGMIM method for the DAPT phase.

The core idea of EGMIM is to introduce structural guidance into the masking process. We hypothesize that by strategically leveraging edge information—a strong indicator of meaningful structures and information-rich local regions—we can steer the model towards learning more powerful and transferable feature representations. This is achieved through a dual mechanism that enhances the pretraining task: it either (1) provides contextual cues by revealing critical structural information within masked regions, or (2) creates targeted reasoning challenges by masking informative local patches. We posit that in low-label scenarios, the quality of features acquired during self-supervised pretraining becomes the primary bottleneck for final performance. By enhancing the model’s capacity to reconstruct informative local structures, EGMIM aims to elevate this performance ceiling, thereby compensating for the scarcity of annotated data during subsequent fine-tuning.

The following subsections detail how this guidance is implemented, first by describing the generation of the Edge-Guided Mask (EGM), and then by justifying the selection of the edge detector.

#### 3.4.1. Generation of the Edge-Guided Mask

Each image $x\in R^{H\times W\times 3}$ in the pretraining dataset is a tensor of height H and width W. Our goal is to generate a binary mask $M_{EGM}\in {\left\{0,1\right\}}^{H\times W}$, where 0 denotes a masked (removed) pixel and 1 denotes a visible pixel. The method modifies a standard SimMIM mask $M_{SimMIM}^{R}$ by incorporating edge-guided cues. Here, the superscript R=0.6 denotes the mask ratio, which is the proportion of pixels to be masked in an input image. SimMIM typically uses a large default window size $W_{p}=32$ for generating its random mask.

(1) Edge-Window Extraction: First, we detect edges from the input image using an unsupervised method, producing a binary edge map $E\in {\left\{0,1\right\}}^{H\times W}$. To align these pixel-level edges with our masking grid, we apply a max-pooling operation with a kernel (and stride) size equal to the small edge-window size w. This yields a coarsened edge indicator map $M_{ew}$:

(2) $$M_{ew}=P_{w}(E)$$

where $P_{w}(\cdot )$ denotes max-pooling and subsequent upsampling (via repeating) operation to restore the map to H×W dimension. Each activated w×w block in $M_{ew}$ is defined as an edge-window, representing a local region likely containing structural boundaries.

**Figure 6:**
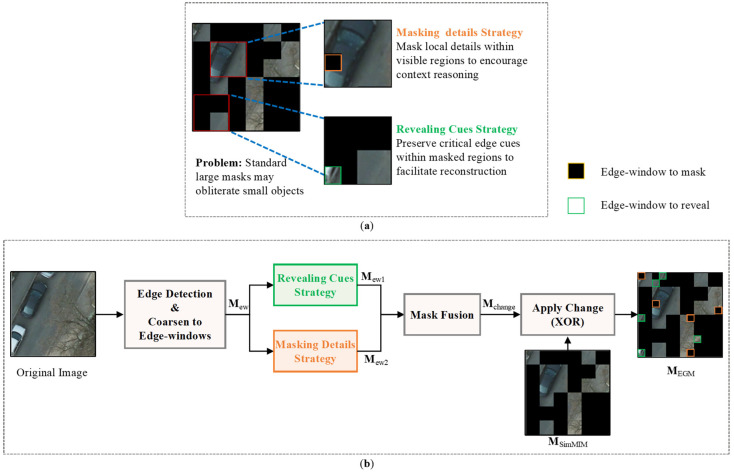
Illustration of the Edge-Guided Masking strategy. (**a**) The core ideas of modifying large-window masking. (**b**) The pipeline for generating the Edge-Guided Mask.

(2) Strategic Selection of Edge-Windows: We define two complementary strategies to sample edge-windows, guided by the original mask $M_{SimMIM}^{R}$, as illustrated in Figure 6a.

(a) Revealing Cues Strategy in Masked Regions: The first strategy aims to provide partial visual cues by unmasking selected edge-windows located within areas that were originally masked by $M_{SimMIM}^{R}$. The target number of edge-windows to unmask, ρ, is determined by a hyperparameter r:

(3) $$\rho =\left\lceil r\cdot H\cdot W/w^{2}\right\rceil$$

where r is the proportion of edge-window areas to unmask, and $\left\lceil \cdot \right\rceil$ denotes the ceiling function. The set of candidate locations within the masked region is obtained via element-wise multiplication (Hadamard product, denoted by ⊙):

(4) $$S_{1}=\left\{locations\;of\;w\times w\;blocks\;in\;M_{ew}⊙\left(1-M_{SimMIM}^{R}\right)\right\}$$

To ensure the sampling process is always valid, the actual number of windows to unmask, $N_{sample1}$, is adaptively set to the minimum of the target number and the available candidates:

(5) $$N_{sample1}=min\left(\rho ,\left|S_{1}\right|\right)$$

where $\left|S_{1}\right|$ denotes the cardinality (size) of the set. This ensures that even if the number of detected edge-windows in the masked region is less than the target (i.e., $\left|S_{1}\right|<\rho$), the algorithm adapts seamlessly by sampling all available candidates. The final set of windows to be revealed is then

(6) $$M_{ew1}=Sample\left(S_{1},N_{sample1}\right)$$

where Sample(S,q) denotes randomly selecting q blocks from set S. $M_{ew1}$ provides partial visual cues (e.g., object boundaries or non-semantic edges) to facilitate the reconstruction of the masked content.

(b) Masking Details Strategy in Visible Regions: The second strategy increases task difficulty by masking selected edge-windows within areas originally visible. The target number ρ is identical. The candidate set is:

(7) $$S_{2}=\left\{locations\;of\;w\times w\;blocks\;in\;M_{ew}⊙M_{SimMIM}^{R}\right\}$$

Similarly, the actual number of windows to mask is controlled by the availability of candidates:

(8) $$N_{sample2}=min\left(\rho ,\left|S_{2}\right|\right)$$

(9) $$M_{ew2}=Sample\left(S_{2},N_{sample2}\right)$$

This set $M_{ew2}$ creates challenging local “puzzles”, forcing the model to infer missing central details from surrounding context, akin to image inpainting.

(3) Mask Fusion: The final set of patches to have their mask status flipped is the union of the two selected sets:

(10) $$M_{change}=M_{ew1}\cup M_{ew2}$$

The final EGM is then obtained by applying an element-wise exclusive OR (XOR, denoted by ⊕) operation between the original mask and the change set:

(11) $$M_{EGM}=M_{SimMIM}^{R}⊕M_{change}$$

This operation flips the mask value $\left(0↔1\right)$ precisely for the patches in $M_{change}$, resulting in a mask that strategically preserves edge cues in some masked regions while obscuring details in some visible regions, as illustrated in Figure 6b.

**Property of the Effective Masking Ratio:** A key property of the proposed EGMIM method is its adaptive nature. Since the actual number of sampled edge-windows, $N_{sample1}$ and $N_{sample2}$, is determined per image based on the availability of edge features ($\left|S_{1}\right|$ and $\left|S_{2}\right|$), they are not necessarily equal in a single forward pass. Consequently, the precise ratio of masked pixels in the final $M_{EGM}$ for an individual image may exhibit a slight deviation from the preset ratio. However, this design is a manifestation of the algorithm’s robustness, not a flaw. It ensures the algorithm adapts to the edge content of each individual image. Across the large set of target domain pretraining images and over multiple training epochs, these image-specific deviations cancel each other out. Therefore, the empirical average masking ratio robustly converges to the preset target ratio R in a statistical sense. This stochasticity does not compromise the stability of the pretraining objective but instead equips the model with the ability to adapt to varying image content.

Similar to SimMIM, we used the EGM strategy to reconstruct the images within the masked region. Specifically, the masked image with EGM is fed into the swinV2 backbone to extract features, and the reconstructed image is obtained with a reconstruction decoder the same as that used in SimMIM. The training objective is an L1 loss computed between the reconstructed and original image within the EGM region, formulated as:

(12) $$loss=\frac{1}{\left|\Omega \right|}{\left\|x_{\Omega }-{x^{\prime }}_{\Omega }\right\|}_{1}$$

where $x_{\Omega }$ and ${x^{\prime }}_{\Omega }$ denote the original and reconstructed image within the masked region Ω, and |Ω| represents the total number of pixels in Ω.

#### 3.4.2. The Selection of Edge Detector

In EGMIM, the edge detector is designed to provide unsupervised structural priors for MIM. Its primary function is to serve as an efficient “attention guidance signal generator”, pinpointing local regions rich in structural information (e.g., areas with pronounced gradients or textures). This guides the model to enhance its capability to represent complex and fine-grained details—a common weakness of standard MIM methods. Consequently, the design inherently tolerates non-semantic boundaries detected by the edge operator, as such regions themselves constitute valuable local contexts for learning. Moreover, in the extreme case where the edge detector fails to capture any valid edges (e.g., in low-contrast or homogeneous texture regions), the EGMIM algorithm naturally degenerates to the standard SimMIM. In this scenario, the two-phase framework remains intact, ensuring a stable performance lower bound. It is worth noting that for high-resolution remote sensing imagery, which typically contains abundant edge details, this extreme case seldom occurs. More commonly, a sufficient number of edge-window candidates are produced, from which a subset is randomly selected during pretraining.

Based on the above analysis, we systematically evaluated edge detectors from different technical paradigms:

(1) Learnable edge detectors: Incorporating a trainable edge detection network (within the framework) would require pixel-level edge annotations from the target domain for supervised training. This directly conflicts with our objective of minimizing annotation dependency, and is therefore considered infeasible in our framework.

(2) Off-the-shelf edge detectors: Such detectors [53,54] are typically trained on natural images. Their performance heavily depends on the training data distribution. When applied to diverse remote sensing target domains, significant domain shift may lead to unstable and unpredictable performance, failing to provide reliable guidance.

(3) Classical unsupervised edge detectors: Training-free unsupervised detectors, which rely solely on low-level image features, are naturally invariant to domain shifts and require no annotation. Among these, we further considered computational efficiency and guidance quality. For instance, super-pixel segmentation algorithms (e.g., SLIC [55]) tend to produce an excessive number of contours, many corresponding to subtle or semantically insignificant textures. This not only dilutes attention and reduces guidance efficacy but also incurs non-negligible computational overhead. In contrast, the classical Canny edge detector achieves a favorable balance in terms of efficiency, controllability, and cross-domain robustness. It is computationally lightweight, adding negligible overhead during the DAPT phase. Its hyper-parameters are intuitive and easy to tune, allowing rapid calibration to extract salient structural responses for different target datasets. As a gradient-based low-level operator, it delivers stable performance across diverse remote sensing domains. Therefore, Canny was selected as the default edge detector in EGMIM for this work.

It should be noted that Canny serves as an effective and empirically validated instantiation here, but it is not the only viable option. In principle, any detector satisfying the requirements of being unsupervised, domain-robust, efficient, and capable of providing salient structural priors could be integrated into the EGMIM framework.

## 4. Experiments

### 4.1. Experimental Setup

#### 4.1.1. Target Domain Datasets

Four remote sensing datasets were selected as target domain datasets to verify the effectiveness of our method respectively. All the images in a target domain dataset are sliced into patches of size 512 without overlapping. Details for each dataset are listed below:

(1) ISPRS Potsdam [56]: a high-resolution (0.05 m) benchmark published by ISPRS Commission WG II/4, containing six semantic classes: cars, impervious surfaces, buildings, trees, low vegetation, and clutter. In our experiments, we utilize only the RGB bands of this dataset while excluding the clutter category during training and validation, following established research protocols. The dataset is partitioned into 24 training images and 14 validation images according to the OpenMMLab convention, which are then processed into non-overlapping 512 × 512 pixel patches. This yields 2904 training patches and 1694 validation patches.

(2) ISPRS Vaihingen [56]: this dataset, acquired over Vaihingen, Germany with a ground sampling distance of 0.09 m, comprises three spectral bands: near-infrared, red, and green. It shares the same six-category labeling scheme as the Potsdam dataset, with the clutter category excluded during training and validation. Following the official dataset division, the 16 training images and 17 validation images were processed into non-overlapping 512 × 512 pixel patches, resulting in 210 training patches and 249 validation patches.

(3) LandCover.Ai [52]: this dataset is an aerial image dataset captured in Poland and central Europe with a resolution of 0.25 m and 0.5 m. The dataset includes five categories: roads, buildings, trees, water, and background. In our experiment, the officially recommended training and validation sets were combined as the training set, and the recommended test set was used as the validation set. We obtained a training set of 9087 images and a validation set of 1587 images.

(4) The Gaofen Image Dataset (GID) [57]: it comprises four-band imagery (near-infrared and RGB) with a 4 m resolution. For our experiments, we utilize only the RGB bands and focus on the coarsely categorized subset containing six land cover classes: forest, water, building, farmland, meadow, and unlabeled “other” regions (excluded from training and validation). All images are processed into non-overlapping 512 × 512 pixel patches, with an 8:2 random split yielding 21,840 training patches and 5460 validation patches.

#### 4.1.2. Implementation Details

The experimental environment for this study consisted of a server equipped with four NVIDIA Tesla V100-32G GPUs (NVIDIA Corporation, Santa Clara, CA, USA), utilizing CUDA 11.3 and PyTorch 1.12.1.

For both the GPPT and DAPT phases, a consistent configuration was employed across all four GPUs, with a total batch size of 512. The AdamW optimizer was adopted with a fixed learning rate of 1 × 10^−4^, betas $\left(\beta_{1},\;\beta_{2}\right)\;=\;\left(0.9,\;0.999\right)$, and a weight decay of 0.05. Each pretraining phase, as detailed in Table 2, was conducted for a sufficient number of iterations to ensure the convergence of the reconstruction loss. To accurately isolate and evaluate the intrinsic efficacy of the pretraining methodology—independent of potential gains from data augmentation—we deliberately excluded any intensity-altering image augmentations. Data augmentation was strictly limited to geometric transformations, including random horizontal/vertical flipping and random scaling.

During the DAPT phase, the proposed EGMIM was implemented with two key hyperparameters: an edge-window size w of 16 and an edge-window ratio r of 0.15. Edge maps were generated using the Canny detector from the scikit-image library. Prior to edge detection, a Gaussian smoothing filter with a standard deviation of 2 was applied to the input images to suppress noise while preserving salient structural information inherent in high-resolution RSIs. All other parameters of the Canny detector were maintained at their default values.

In the subsequent finetuning step for semantic segmentation, the input image size was set to 512 × 512 (different from pretraining). To accommodate this change, the shift window size of the SwinV2-B backbone was adjusted from 12 to 16. The consequent alterations in RPB were compensated for by a small, randomly initialized multi-layer perceptron, while all other parameters of the SwinV2-B backbone were initialized with the weights obtained from the DAPT phase. Finetuning was performed on a single GPU with a batch size of 2. For the backbone parameters, the AdamW optimizer was used in conjunction with a cosine annealing learning rate schedule. The initial learning rate was set to 1.95 × 10^−5^ for the parameters in the 24th transformer layer, with a layer-wise decay factor of 0.75 applied to the parameters in progressively shallower layers. The weight decay was set to 0.05. For the segmentation decoder, the SGD optimizer was employed with a momentum of 0.9, a weight decay of 5 × 10^−4^, and a polynomial learning rate scheduler (power = 1) that decayed linearly from an initial rate of 0.01 to zero. To exclude any potential benefits from intensity-based augmentations, data augmentation was restricted solely to geometric transformations—specifically, horizontal/vertical flipping and 90-degree rotations, each applied with a probability of 50%—thereby ensuring a precise evaluation of the representations learned during pretraining.

To ensure the statistical robustness of our experiments, we adopted a strictly controlled experimental protocol across all studies. First, to eliminate performance variance arising from the random selection of labeled samples—a major concern in low-label regimes—we first fixed the order of the entire training set. For each labeling ratio experiment (e.g., 1/100, 1/32), we consistently selected the first corresponding proportion of samples from this ordered list. This guarantees that the training set for a higher labeling ratio is a strict superset of that for a lower ratio, isolating the effect of annotation quantity. Second, for the key experiments evaluating our proposed frameworks, we conducted three independent runs. Each run was performed with a different, pre-specified random seed controlling all stochastic processes (parameter initialization for segmentation decoders, data augmentation sequences, etc.). Third, for the hyperparameter ablation study and variant analysis, which involved screening a large number of configurations, results are from a single but controlled run (using the fixed data subset) to efficiently illustrate comparative trends. For a detailed description of the protocol for comparing with state-of-the-art (SOTA) methods, please refer to Section 4.5.

#### 4.1.3. Evaluation Metrics

The category with the highest probability of the segmentation network’s prediction is regarded as the prediction result for each pixel in an image. As we focused on the semantic segmentation of RSIs, the mean F1-score (mF1) and mean Intersection over Union (mIoU) for all the categories were used as the assessment metrics for the overall performance of the segmentation network.

### 4.2. Comprehensive Comparison of Pretraining Frameworks

We performed different pretraining frameworks with the Potsdam dataset, and the segmentation model was finetuned under different labeling ratios in the training set. Then, three other datasets were used to further verify the superiority of our proposed pretraining framework.

We begin by defining the notation used to describe the pretraining process. Based on insights from existing studies, we identified three key factors that significantly influence pretraining performance: the dataset used, the training algorithm, and the number of training iterations. Accordingly, we denoted the process of pretraining a backbone network on a dataset **DATA** using method **ALGORITHM** for **NUM** epochs as ${\mathrm{DATA}}_{\mathrm{ALGORITHM}}^{\mathrm{NUM}}$. Since SimMIM is the default algorithm used for pretraining SwinV2 in our study, we abbreviate the pretraining process as ${\mathrm{DATA}}^{\mathrm{NUM}}$ when using SimMIM. Pretraining with the EGMIM method is denoted as ${\mathrm{DATA}}_{EGMIM}^{\mathrm{NUM}}$. Prior research suggests that increasing the number of iterations generally improves performance. Therefore, in our experiments, we ensure sufficient training epochs until the training loss converges. The default number of epochs used for pretraining on each dataset, along with the corresponding notation, is provided in Table 2.

We represent the two-phase pretraining framework by combining the pretraining processes listed in Table 2 in the form of GPPT+DAPT. For consistency, single-phase pretraining strategies are also expressed using this unified notation, where “SKIP” is used to denote the absence of either the GPPT or DAPT phase. As an example, the notation for a two-phase pretraining process using HRGC for GPPT and Potsdam for DAPT is illustrated in Table 3.

#### 4.2.1. Results on the Potsdam Dataset

Table 4 presents a comprehensive benchmarking of semantic segmentation performance under varying labeling ratios on the Potsdam dataset, comparing five representative pretraining frameworks.

First, the results challenge a simplistic view of pretraining data superiority. Within single-phase frameworks, ${\mathrm{IN}}^{800}+\mathrm{SKIP}$ consistently outperforms ${\mathrm{HRGC}}^{400}+\mathrm{SKIP}$ across all labeling ratios, despite ImageNet-22K containing no remote sensing imagery. This finding challenges the conventional assumption that simply replacing natural images with large-scale RSIs for VFM pretraining leads to performance gains. Meanwhile, $\mathrm{SKIP}+{\mathrm{Pot}}^{800}$, which employs 800 epochs of DAPT (requiring 4× more GPU hours than two-phase frameworks in the DAPT phase) to compensate for the absence of GPPT, achieves the lowest performance under full labeling. This indicates that the GPPT phase significantly enhances both the training efficiency of DAPT and final accuracy of the network.

Second, our experiments decisively demonstrate the superiority of the two-phase GPPT+DAPT paradigm. All two-phase frameworks significantly outperform their single-phase counterparts. Crucially, the comparison between the two two-phase implementations is revealing: ${\mathrm{HRGC}}^{400}+{\mathrm{Pot}}^{200}$ (our GPPT+DAPT) consistently surpasses ${\mathrm{IN}}^{800}+{\mathrm{Pot}}^{200}$ (ImageNet+DAPT) across all labeling ratios. This provides direct, empirical evidence that starting the two-phase process with a domain-native foundation (GPPT) yields a more effective final model than starting from a generic natural image foundation.

Third, the proposed framework excels in low-label regimes. ${\mathrm{HRGC}}^{400}+{\mathrm{Pot}}^{200}$ achieves the highest accuracy, with its advantage being most pronounced under the stringent 1/100 labeling ratio. Notably, the addition of the DAPT phase to the HRGC-pretrained backbone (${\mathrm{HRGC}}^{400}+{\mathrm{Pot}}^{200}$ vs. ${\mathrm{HRGC}}^{400}+\mathrm{SKIP}$) brings a dramatic improvement of up to 6.74% mF1-score, highlighting DAPT’s critical role in adapting the general-purpose features to the specific target domain, especially when labels are scarce.

In summary, the results on Potsdam establish a clear hierarchy: GPPT+DAPT (Ours) > ImageNet+DAPT > Single-phase strategies. This forms the cornerstone of our argument for the proposed pretraining framework.

Furthermore, the comprehensive comparison on Potsdam establishes the GPPT+DAPT framework as the superior paradigm, decisively outperforming the alternative ImageNet+DAPT baseline. Based on this conclusive finding, the focus of the subsequent experiments on Vaihingen, LandCover.Ai, and GID shifts from rediscovering the optimal framework to investigating two critical and practical questions: (1) How essential is the DAPT phase when the GPPT foundation is applied to target domains with distinctly different characteristics (e.g., spectral bands, resolution)? (2) Does the performance gain hold consistently across these diverse domains? Therefore, in Section 4.2.2, Section 4.2.3 and Section 4.2.4, we adopt the established optimal starting point—the HRGC-pretrained model—as the common baseline, and concentrate on ablating the necessity and effectiveness of the subsequent DAPT phase under various domain shift scenarios.

#### 4.2.2. Results on the Vaihingen Dataset

The Vaihingen dataset, with its IRRG band configuration, presents a substantial spectral domain shift compared to the RGB-based HRGC pretraining corpus. This makes it an ideal testbed to examine whether the performance gain from the DAPT phase, as observed on Potsdam, generalizes to scenarios with significant low-level feature distribution differences.

As shown in Table 5, incorporating the DAPT phase (${\mathrm{HRGC}}^{400}+{\mathrm{Vai}}^{800}$) consistently outperforms the single-phase baseline (${\mathrm{HRGC}}^{400}+\mathrm{SKIP}$) across all labeling ratios, achieving mF1 improvements ranging from +3.04% to +2.36%. This result robustly validates the critical role of DAPT in bridging spectral domain gaps. Even after acquiring general remote sensing knowledge from HRGC, a dedicated adaptation phase on the target domain’s unlabeled data is indispensable for optimal performance. Crucially, this persistent performance gap, which remains even under full supervision, indicates that the fundamental feature distribution shift caused by spectral differences cannot be fully compensated for by supervised fine-tuning alone. It must be addressed at the pretraining phase through domain adaptation.

#### 4.2.3. Results on the LandCover.Ai Dataset

The LandCover.Ai dataset is included as a subset in our HRGC pretraining corpus. Consequently, the inherent domain shift between GPPT and this target domain is minimized, providing a setting to evaluate the DAPT phase’s effect when the initial feature mismatch is less severe.

Results in Table 6 show that even under this condition, the DAPT phase (${\mathrm{HRGC}}^{400}+{\mathrm{LC}}^{100}$) yields consistent, albeit smaller, improvements over the baseline (${\mathrm{HRGC}}^{400}+\mathrm{SKIP}$), with gains from +0.49% to +0.61%. This demonstrates that DAPT’s utility extends beyond correcting large distributional shifts; it also performs fine-grained feature specialization and regularization on the target data, thereby consistently boosting performance.

#### 4.2.4. Results on the GID

The GID exhibits a significant resolution gap (4 m) compared to the high-resolution imagery (0.1–0.5 m) in HRGC. This tests the adaptability of the pretraining framework to variations in spatial scale and object appearance granularity.

As presented in Table 7, the DAPT phase (${\mathrm{HRGC}}^{400}+{\mathrm{GID}}^{20}$) again delivers solid improvements over the single-phase strategy (${\mathrm{HRGC}}^{400}+\mathrm{SKIP}$), with mF1 gains consistently exceeding +0.65% across all extremely low to full labeling ratios. This confirms that the DAPT phase is highly effective in adapting a high-resolution-pretrained model to a lower-resolution target domain, enabling the model to recalibrate its feature extractor to the new scale.

### 4.3. Effectiveness of EGMIM

The preceding sections have validated the GPPT+DAPT framework as a superior pretraining paradigm. Within this framework, we further propose EGMIM to enhance the DAPT phase. This section evaluates whether incorporating structural (edge) priors into the masking strategy can bring additional gains, particularly in the low-label regimes central to this work.

As shown in Table 4, Table 5, Table 6 and Table 7, EGMIM provides consistent and most substantial improvements under extreme low-label conditions. For instance, on Potsdam at the 1/100 labeling ratio, it delivers a +0.61% mF1 gain over the base DAPT (${\mathrm{HRGC}}^{400}+{\mathrm{Pot}}^{200}$). This aligns perfectly with our design motivation: when supervisory signals are scarce, the quality of self-supervised features becomes paramount. By prioritizing the reconstruction of edge-rich regions, EGMIM fosters representations with a stronger inherent capacity for discriminating fine-grained structures, which is critical for segmentation with limited labels.

As the labeling ratio increases, the relative advantage of EGMIM diminishes, sometimes matching or slightly underperforming the base DAPT. This trend corroborates our hypothesis: when abundant annotated data is available during finetuning, the demand for exceptionally powerful pretrained features is less critical. Thus, EGMIM’s principal value is in mitigating the low-label performance bottleneck, precisely where pretraining matters most.

**Clarification on the Role of Edge Guidance**. It is important to emphasize that the edge information in EGMIM serves solely as a spatial prior to guide the masking process during self-supervised pretraining. It is not used as an explicit loss or constraint in the subsequent supervised finetuning step. Therefore, the gains should not be interpreted as EGMIM directly “sharpening” segmentation boundaries of specific objects. Instead, by forcing the model to reconstruct regions rich in structural details, EGMIM fosters the learning of more transferable feature representations. This enhanced representation inherently possesses a stronger capacity for distinguishing fine-grained structures, which manifests as an overall improvement in segmentation accuracy, particularly when labeled data is scarce.

### 4.4. Ablation Studies

All ablation studies were conducted on Potsdam dataset.

#### 4.4.1. Impact of GPPT Configurations

As shown in Table 8, we conducted an ablation study to dissect the impact of configurations within the GPPT phase on the final performance of our proposed GPPT+DAPT framework. The results yield two critical insights into designing an effective GPPT phase:

First, the sufficiency of training is paramount. Comparing ${\mathrm{HRGC}}^{400}+{\mathrm{Pot}}^{200}$ with ${\mathrm{HRGC}}^{100}+{\mathrm{Pot}}^{200}$ reveals that a more thoroughly trained GPPT model (400 epochs vs. 100 epochs) consistently leads to better downstream performance across all labeling ratios. This underscores that a robust and converged feature representation from the GPPT phase forms a stronger foundation for subsequent domain adaptation.

Second, the richness and diversity of the pretraining data are key factors. The configuration ${\mathrm{Inria}}^{800}+{\mathrm{Pot}}^{200}$, which uses a subset of the HRGC corpus for GPPT, is consistently outperformed by ${\mathrm{HRGC}}^{400}+{\mathrm{Pot}}^{200}$. This indicates that scaling up the diversity and volume of RSIs in the GPPT phase is beneficial for learning transferable general-purpose features.

These findings solidify the design principles for the first phase of our framework: an effective GPPT requires both adequate training iterations and a large, diverse domain-native dataset. This optimized GPPT foundation is crucial for maximizing the gains from the subsequent DAPT phase, especially in data-scarce scenarios.

#### 4.4.2. Impact of EGMIM Hyper-Parameters

The hyper-parameters of EGMIM, specifically the edge-window size w and ratio r, dictate how structural priors are integrated into the self-supervised objective. Analyzing their impact is crucial to understanding the method’s sensitivity and validating its design for low-label regimes.

We evaluate w and r within our established optimal framework, ${\mathrm{HRGC}}^{400}+{\mathrm{Pot}}_{EGMIM}^{200}$. As shown in Table 9, we test $w\in \left\{8,\;16,\;24\right\}$ (all smaller than SimMIM’s default patch size of 32) and $r\in \left\{0.05,\;0.10,\;0.15,\;0.20\right\}$ (below SimMIM’s mask ratio of 0.6). The results reveal several key insights that align with our methodological design:

(1) Controlled local guidance is beneficial. Increasing the edge-window ratio r from 0.05 to 0.15 consistently improves performance under the critical 1/100 labeling ratio (mF1 from 83.62 to 84.85), confirming that explicitly focusing the model on learning from edge-rich local regions is a potent strategy when labeled data is scarce. Performance saturates as r increases further.

(2) The spatial scope of guidance must be balanced. Fixing r=0.15, a moderate window size w=16 yields the best results (84.85% mF1). A smaller window (w=8, 83.04% mF1) may provide too narrow a context, while a larger one (w=24, 83.86% mF1) may dilute the local structural signal. This validates our design choice for a localized guidance mechanism.

(3) The impact is most pronounced in low-label settings. The performance gap between different hyper-parameter settings is largest at the 1/100 labeling ratio and diminishes significantly as more labels become available. This trend strongly supports EGMIM’s core premise: it is specifically designed to compensate for the lack of supervisory signals by leveraging unlabeled structural priors, making it particularly valuable in the low-label scenarios that are the focus of this work.

#### 4.4.3. Experiments on Two Variants of EGMIM

To dissect the individual contributions of the two core strategies in our EGM, we develop and evaluate two variants: EGMIMv1 and EGMIMv2. These variants are designed to isolate the effects of “revealing cues” and “masking details”, respectively, as described below.

(1) GMIMv1 (revealing cues): This variant implements only the strategy of providing partial visual information within masked regions. To maintain the exact global mask ratio R=0.6, we start with a base SimMIM mask with an increased ratio R+r. We then unmask a set of edge-windows selected from this enlarged masked area. Thus, EGMIMv1 tests the benefit of granting the model explicit access to critical edge cues during reconstruction.

(2) EGMIMv2 (masking details): This variant implements only the strategy of enforcing localized context reasoning. It starts from a base SimMIM mask with a decreased ratio R−r. We then mask an additional set of edge-windows selected from the initially visible region. Therefore, EGMIMv2 evaluates the benefit of creating challenging inpainting-like puzzles centered on edges.

In this experiment, we set w=16 and r=0.15 for both variants. This design ensures a controlled comparison where the overall mask ratio remains constant, allowing us to attribute performance differences directly to the distinct learning mechanisms induced by each strategy.

As shown in Table 10, the integrated EGMIM approach consistently outperforms both variants across all labeling ratios. The superior performance of EGMIMv2 over EGMIMv1, particularly under higher label ratios, reveals a fundamental insight: the constraint of reconstructing precisely masked local edge regions in EGMIMv2 forces the model to develop a more powerful and generalizable understanding of local context and geometric structures, which is crucial for dense prediction tasks. In contrast, while EGMIMv1 provides helpful low-level cues for bootstrapping reconstruction, its benefits may saturate as the model increasingly relies on the provided edge information rather than learning to infer it from context.

These results confirm that the two strategies are not merely complementary but operate on different learning principles. EGMIMv1 acts as a form of “guided reconstruction”, reducing the initial learning difficulty by preserving critical signals. EGMIMv2, however, introduces a form of “structured difficulty”, creating targeted, local inpainting challenges that are inherently aligned with the goal of semantic segmentation—precisely understanding object boundaries and fine-grained details. The synergy in the complete EGMIM method arises because the “guided reconstruction” of EGMIMv1 helps stabilize the early stages of learning the challenging “structured difficulty” tasks introduced by EGMIMv2, leading to the most robust and effective feature representation.

In summary, EGMIM’s superiority stems from its algorithmic design that intrinsically balances two learning dynamics. It achieves an equilibrium between exploiting explicit edge cues to ease optimization and imposing structured inpainting challenges to force stronger contextual reasoning. This balance is not a manually tuned hyper-parameter but an emergent property of the unified masking strategy, enabling the model to concurrently learn both fine-grained local representations and their coherent integration into a global context.

### 4.5. Comparison with the State-of-the-Art Methods

To ensure a fair and reproducible comparison with SOTA methods, we adhere to the following protocol: For each SOTA approach, we initialize the model with its officially released pretrained weights and subsequently finetune it under our unified experimental setup—using the same fixed data splits and evaluation metrics as described in Section 4.1. For models without publicly available weights (i.e., RingMo and CtxMIM), we reproduce their pretraining using the HRGC dataset. Regarding segmentation heads, ResNet-based [58] models are paired with DeeplabV3+ [59], while transformer-based models (ViT and swin series) use a lightweight FCN head for consistency.

The comprehensive comparison results are presented in Table 11. Our proposed two-phase framework (Ours1), despite its conceptual simplicity, consistently outperforms most SOTA methods across all labeling ratios on both mIoU and mF1 metrics. The incorporation of EGMIM (Ours2) further amplifies this advantage, particularly in low-label regimes. At the extremely challenging 1/100 ratio, Ours2 surpasses the best competitor by 1.91% in mIoU and 1.27% in mF1. The performance gains, though more modest at higher labeling ratios, remain consistent. This significant and statistically robust gap (as indicated by the low standard deviation of our results) underscores that many existing pretraining paradigms are optimized for high-data scenarios, often overlooking the critical challenge of limited annotations. The strong performance of our method under data scarcity validates its practical utility, for instance, in providing a more reliable initial teacher model for subsequent semi-supervised learning pipelines, thereby mitigating error propagation from noisy pseudo-labels.

The results also reveal an interesting trend: at lower annotation ratios, ViT-based models often underperform their ResNet-50 counterparts. We attribute this to factors beyond pretraining: (1) Standard ViT architectures lack the inherent inductive bias for multi-scale feature representation compared to hierarchical CNNs like ResNet, and (2) their substantially larger parameter counts (e.g., ViT-B: 86M vs. ResNet-50: 25M) make them more prone to overfitting when data is scarce. Although swin-based models—with their hierarchical design—generally outperform standard ViT architectures, their performance shows high variance across different pretraining studies, highlighting the sensitivity of transformer-based models to pretraining efficacy.

**Figure 7:**
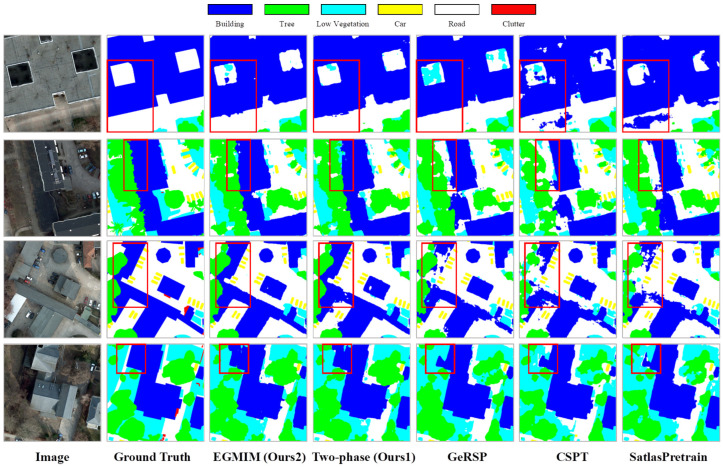
Qualitative comparison of segmentation results on Potsdam validation set under 1/16 labeling ratio.

We further provide a qualitative comparison of segmentation results under the 1/16 labeling ratio in Figure 7. Visually, predictions from our two-phase framework (Ours1) and its EGMIM-enhanced variant (Ours2) exhibit greater semantic consistency and larger coherent regions compared to competing methods. For instance, areas such as large buildings or vegetation patches are more completely and accurately recognized by our methods, with fewer spurious misclassifications. This aligns with our quantitative findings in Table 11 and suggests that the representations learned through our GPPT+DAPT paradigm lead to more robust and context-aware features, which are particularly beneficial when annotated data is scarce. While the edge-guided mechanism in EGMIM operates during pretraining and is not explicitly enforced during finetuning, the overall segmentation quality improvement is evident in these holistic visual comparisons.

### 4.6. Efficiency Analysis of the Pretraining Frameworks

As a core part of our comprehensive benchmarking, this section provides a detailed efficiency analysis comparing the proposed framework with all alternative strategies outlined in Section 4.2.1. We focus on two key metrics: wall-clock training time and GPU memory footprint, which offer a complete picture of the framework’s practicality.

The analysis is based on experiments on the Potsdam dataset (100% labeling ratio). To reflect realistic research and development scenarios, GPPT and DAPT times are reported in 4-GPU parallel computation hours, while finetuning time is reported in single-GPU computation hours (see Section 4.1.2). It is crucial to note that GPPT, as a one-time, general-purpose foundational model pretraining, is a reusable asset. Once obtained, the pretrained backbone can serve as the starting point for DAPT or any downstream task. Therefore, the marginal time cost for applying our framework to a new target domain considers only the DAPT and finetuning phases. From the comparative results summarized in Table 12, we draw several key conclusions that highlight the efficiency–accuracy trade-offs of different strategies:

(1) The high value of a reusable, domain-native foundation. The DAPT phase in our framework (${\mathrm{HRGC}}^{400}+{\mathrm{Pot}}^{200}$), benefiting from the high-quality initialization provided by GPPT, converges rapidly on target domain data in just 5 h. In contrast, the baseline $\mathrm{SKIP}+{\mathrm{Pot}}^{800}$—which lacks a strong pretrained foundation—requires a protracted 18 h DAPT phase yet achieves inferior performance (mF1 87.48%). This clearly demonstrates that the upfront investment in GPPT is not an invalid cost; rather, it drastically improves the sample efficiency and convergence speed of the subsequent adaptation phase.

(2) Superior efficiency–accuracy trade-off. Our framework establishes a superior point on the efficiency–accuracy compared to all alternative strategies. When benchmarked against end-to-end supervised training initialized from ImageNet (${\mathrm{IN}}^{800}+\mathrm{SKIP}$), our approach delivers a significant performance gain of +1.55% mF1 for a marginal time investment of approximately 10 h. Compared to the single-phase GPPT baseline (${\mathrm{HRGC}}^{400}+\mathrm{SKIP}$), the introduction of the DAPT phase requires only an additional 5 h but yields a substantially larger improvement of +2.48% mF1, demonstrating the high cost-effectiveness of the two-phase design. Most critically, in a direct comparison with the alternative two-phase paradigm (${\mathrm{IN}}^{800}+{\mathrm{Pot}}^{200}$), our framework operates at the same marginal cost of 10 h yet achieves a superior +1.06% mF1 gain. This result definitively shows that the performance advantage is attributable to the domain-native GPPT foundation, not merely to the two-phase structure itself.

(3) Minimal Memory Overhead. The memory usage (last row of Table 12) aligns with our experimental setup. The proposed EGMIM modifies only the masking strategy of SimMIM; edge detection is a preprocessing step, introducing no additional GPU memory overhead during training. Thus, memory consumption for GPPT and DAPT remains at 24.7 GB/GPU, identical to standard SimMIM. Finetuning with a lightweight head further reduces memory demand to 7.7 GB/GPU.

In summary, this efficiency benchmarking that is integral to our study demonstrates that our GPPT+DAPT framework does not merely increase computational budget. By amortizing the cost of a reusable, domain-expert foundation (GPPT) and enabling an efficient, fast-converging DAPT phase, it achieves a more favorable efficiency–accuracy optimum. This provides a practical and efficient pathway to high performance in label-scarce scenarios.

## 5. Discussion

The experimental results in Section 4 demonstrate the clear superiority of the GPPT+DAPT framework. To gain deeper insights into why this framework outperforms alternatives and how each phase contributes to the final representation, we conduct a series of diagnostic analyses on the backbones pretrained in the Potsdam experiment, providing a mechanistic explanation for the empirical observations.

### 5.1. Analysis of Pretrained RPB Patterns

**Figure 8:**
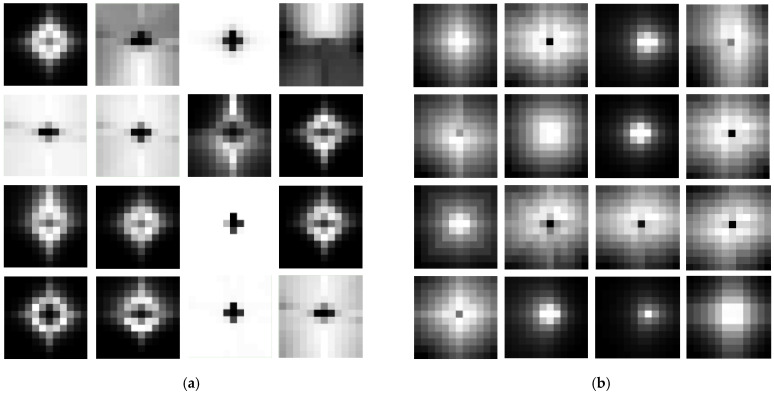
RPBs (with query point in the image center) in the 22nd transformer layer of the pretrained backbone, dark colors are low attention zones; light colors are high attention zones. (**a**) RPBs pretrained with ImageNet; (**b**) RPBs pretrained with ${\mathrm{HRGC}}^{400}$ and ${\mathrm{HRGC}}^{400}+{\mathrm{Pot}}^{200}$.

The RPB in Equation (1) models spatial relationships between tokens to adjust attention weights. To understand the inherent spatial priors learned during pretraining, we visualize the RPB of the last layer in stage 3 of the swinV2-B backbone. This stage contains 16 attention heads, each yielding a unique RPB pattern, as shown in Figure 8. The analysis reveals two key insights that mechanistically explain the superiority of our framework:

(1) **Domain-specific attention characteristics**. RPB patterns from models pretrained on ImageNet-22K differ fundamentally from those pretrained on RSIs. While several heads in the ImageNet-pretrained model (e.g., heads 3, 5, 6, 11, 15, 16) attend to global regions, all 16 heads in the RSI-pretrained models exhibit a strong focus on local neighborhoods around the query token. This aligns with a core domain difference: natural images often center on dominant objects requiring global context, whereas RSIs are composed of localized, semantically coherent geographic entities, resonating with Tobler’s First Law of Geography. Despite swinV2-B’s capacity for global attention, RPB visualization confirms strong locality priors emerge from RSI pretraining.

(2) **Phase-specific pattern adaptation**. While the RPB distributions from GPPT and DAPT appear visually similar, quantitative differences exist in the magnitude of their position encodings. This indicates a functional specialization: GPPT establishes the fundamental, topology-aware geometry of spatial attention, which is domain-native. Subsequently, DAPT performs a targeted calibration of these patterns, refining their numerical parameters for the specific target dataset without altering the core attention structure. This efficient adaptation aligns with the parameter evolution analysis in Section 5.4.

### 5.2. Characteristics of Pretrained Average Attention Score

The final attention score, formalized in Equation (1), integrates both content affinity and the learned RPB. To analyze the spatial patterns across all attention heads, we computed an attention heatmap by averaging scores from all heads for a given query, i.e.,

(13) $$average\;attention\;score=\frac{1}{h}\sum_{i=1}^{h}{attention\;score}_{i}\left(K_{i},Q_{i}\right)$$

**Figure 9:**
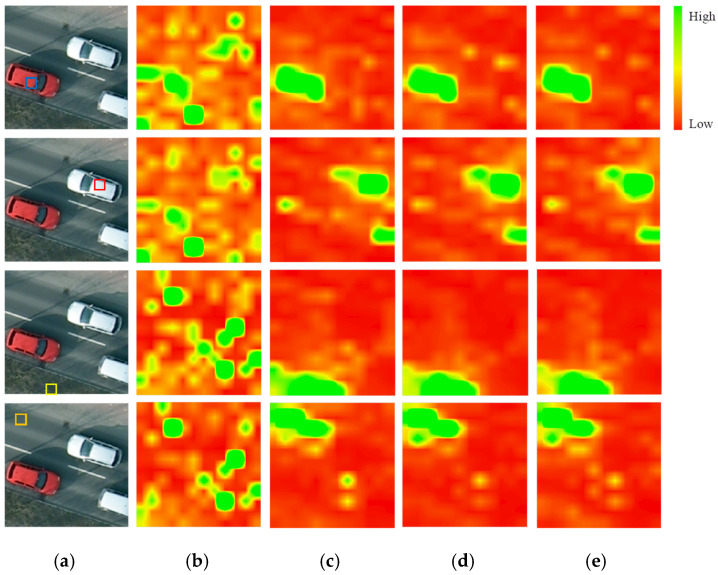
Comparative visualization of attention heatmaps under different pretraining configurations. (**a**) Original aerial image with query locations marked by colored boxes; (**b**) ImageNet pretraining (${\mathrm{IN}}^{800}$); (**c**) ${\mathrm{HRGC}}^{400}$ pretraining; (**d**) ${\mathrm{HRGC}}^{400}+{\mathrm{Pot}}^{200}$ pretraining; (**e**) ${\mathrm{HRGC}}^{400}+{\mathrm{Pot}}_{EGMIM}^{200}$ pretraining. All heatmaps are interpolated to the original image size and visualized using root-mean-square normalization with one standard deviation. The colored boxes in (**a**) mark the query locations used for generating the attention heatmaps.

Figure 9 contrasts heatmaps for four query locations under different pretraining workflows. Column (b), from the ImageNet-22K-pretrained model (${\mathrm{IN}}^{800}$), exhibits fragmented and semantically uninterpretable patterns. In stark contrast, columns (c)–(e)—models involving HRGC pretraining—show spatially coherent and semantically meaningful attention, strongly clustered around features similar to the query (e.g., car queries focus on cars). This yields two principal insights supporting our framework’s efficacy:

(1) **Essential inductive bias from RSI Pretraining**. The sharp, class-aware attention from HRGC-based models (${\mathrm{HRGC}}^{400}$) demonstrates that large-scale remote sensing data provides a fundamental, domain-native clustering prior. This prior, absent in natural image pretraining, is crucial for learning transferable features that enable higher accuracy with limited labels, directly explaining the performance gap observed in Section 4.2.1.

(2) **Refined Attention through DAPT**. While the core spatial distribution remains consistent after HRGC pretraining, the DAPT phase (${\mathrm{HRGC}}^{400}+{\mathrm{Pot}}^{200}$) and its enhanced variant (${\mathrm{HRGC}}^{400}+{\mathrm{Pot}}_{EGMIM}^{200}$) calibrate the attention response, enabling better alignment with the target domain’s feature distribution. This refinement, rather than a complete overhaul, underpins the consistent performance gains of the DAPT phase.

**Figure 10:**
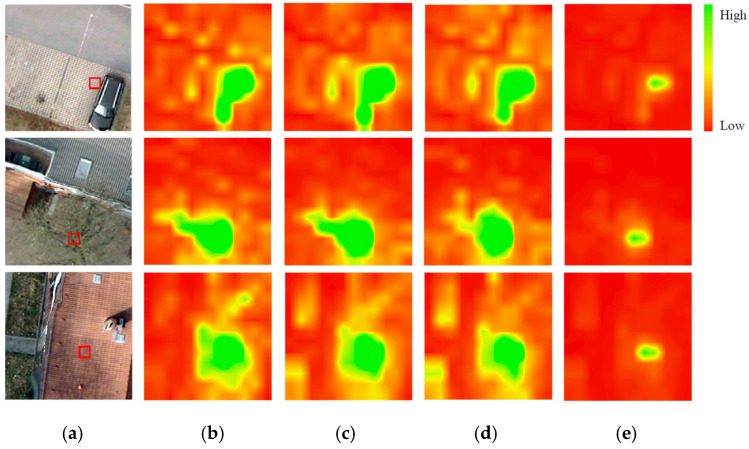
Comparative visualization of attention heatmaps under different pretraining workflows. (**a**) Original aerial image with query locations marked by red boxes; (**b**) ${\mathrm{HRGC}}^{400}$ pretraining; (**c**) ${\mathrm{HRGC}}^{400}+{\mathrm{Pot}}^{200}$ pretraining; (**d**) ${\mathrm{HRGC}}^{400}+{\mathrm{Pot}}_{EGMIM}^{200}$ pretraining; (**e**) enhanced visualization of (**d**) using max-min stretching to emphasize salient regions. The red boxes in (**a**) mark the query locations used for generating the attention heatmaps.

Further analysis on large, homogeneous objects (Figure 10) reinforces the localized nature of the attention mechanism. For queries within expansive, uniform regions (e.g., building roofs, roads), the attention heatmaps remain concentrated in the immediate vicinity of the query token rather than dispersing across the entire object. Crucially, this occurs even when the query-key affinity could be similar over a large area, providing compelling evidence that the learned RPB is the dominant factor shaping the attention pattern. This confirms that the pretrained RPB imposes a strong spatial prior for local interactions, which is directly visualized in the RPB matrices themselves (Section 5.1). Ultimately, this bias for building features from a spatially proximate context is fundamental to RSI understanding, as it aligns with the inherent spatial coherence of geographic entities.

### 5.3. Visualization of Feature Distribution Shifts

**Figure 11:**
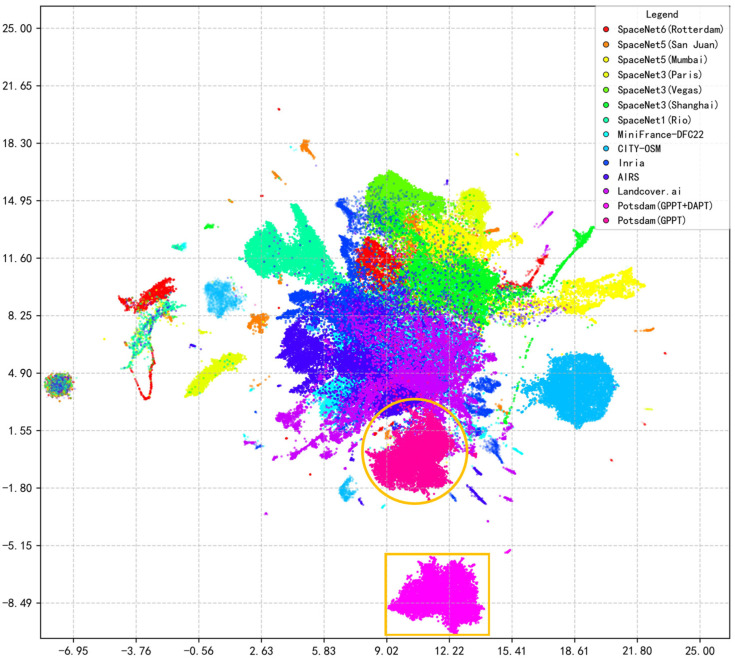
Visualization of the feature distributions of selected samples from the GPPT data and the Potsdam target domain. The distribution of Potsdam features encoded using only the GPPT workflow is highlighted with an orange circle, while features processed with the proposed GPPT+DAPT workflow are enclosed in an orange rectangle. Each colored cluster (not enclosed by orange lines) represents a sub-dataset within the GPPT data. Significant domain shifts can be observed between the GPPT data and Potsdam (GPPT), as well as between Potsdam (GPPT) and Potsdam (GPPT+DAPT).

To intuitively understand the representational effects of different pretraining phases, we analyze the feature space geometry. We encode features from selected HRGC sub-datasets and the Potsdam target domain using the backbone pretrained solely on ${\mathrm{HRGC}}^{400}$ and visualize their distributions via UMAP [61], as shown in Figure 11. The visualization reveals that the features learned during GPPT exhibit both internal diversity and a persistent domain gap. Specifically, different sub-datasets within the HRGC corpus occupy distinct yet partially overlapping regions, forming a broad feature distribution. However, a clear separation exists between this GPPT-learned distribution and the features extracted from the Potsdam target domain by the same GPPT-only model (marked by the orange circle), visually confirming the inherent domain shift that cannot be fully bridged by GPPT alone.

The necessity and impact of the DAPT phase are directly evidenced by the significant shift in feature distribution. As shown in Figure 11, the feature distribution of the Potsdam dataset extracted by the model after the complete GPPT+DAPT framework (marked by the orange rectangle) undergoes a pronounced spatial realignment relative to the distribution from the GPPT-only model. This distinct change demonstrates that the DAPT phase facilitates a targeted reconstruction of the feature space, effectively adapting the general-purpose representations from GPPT to better align with the specific characteristics of the target domain. This visual evidence forms a mechanistic link to the empirical performance gains observed in Section 4.2, providing a comprehensive explanation for the effectiveness of the DAPT phase.

### 5.4. Parameter Difference Analysis

**Figure 12:**
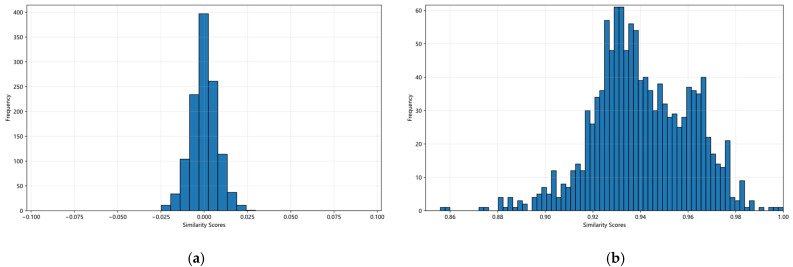
Distribution of parameter group similarity across pretraining strategies. (**a**) Similarity between models pretrained on ImageNet and ${\mathrm{HRGC}}^{400}$. The scores concentrated near zero indicate fundamental divergence, justifying domain-specific GPPT. (**b**) Similarity between the GPPT checkpoint (${\mathrm{HRGC}}^{400}$) and the DAPT-adapted model (${\mathrm{HRGC}}^{400}+{\mathrm{Pot}}_{EGMIM}^{200}$). The high similarity demonstrates that DAPT performs consistent optimization, preserving general features while adapting to the target domain.

In this section, we analyze the parameter differences in the backbone network across three distinct pretraining workflows. To quantitatively assess these differences, we employ a parameter-grouping similarity analysis. Directly comparing individual parameters offers limited interpretability. Therefore, we grouped parameters by their functional units (e.g., treating the query weight matrix within each self-attention module as a distinct group). For any two models under comparison, we computed the cosine similarity between each pair of corresponding functional groups, yielding a set of N similarity scores (where N is the total number of groups). The distribution of these scores, visualized in Figure 12, provides a macroscopic view of the overall similarity or divergence between the two models in the parameter space.

First, Figure 12a compares the backbone parameters pretrained on ImageNet against those pretrained on our large-scale RSI dataset, ${\mathrm{HRGC}}^{400}$. The histogram shows a pronounced concentration of similarity scores near zero (within [−0.025, 0.025]), indicating that the learned parameter sets are fundamentally divergent. This provides direct, parameter-level evidence that pretraining on natural images versus large-scale remote sensing imagery leads to intrinsically different feature representations. It underscores the necessity of RSI-specific GPPT as the first phase, which establishes a domain-relevant parameter foundation distinct from models transferred from the natural image domain.

Conversely, Figure 12b analyzes the parameter evolution within the RSI domain, specifically between the GPPT checkpoint (${\mathrm{HRGC}}^{400}$) and the same model after DAPT (${\mathrm{HRGC}}^{400}+P_{EGMIM}^{200}$). Here, the similarity scores are predominantly high (>0.85), with a peak ranging from 0.92 to 0.98. This demonstrates that the DAPT phase performs a consistent and stable refinement of the existing parameters rather than a complete overhaul. The high similarity confirms that DAPT functions as an adaptive finetuning phase, effectively preserving the beneficial general features learned during GPPT.

Crucially, when juxtaposed with the substantial feature distribution shift observed in Figure 11, these parameter-level findings reveal a key insight: Even subtle, targeted parameter adjustments during DAPT (as evidenced by high overall similarity) are sufficient to induce significant and beneficial changes in the feature representation space for the target domain. This high sensitivity and efficacy underscore the indispensable role of DAPT in bridging the domain gap between the generic pretraining data and specific target applications, ultimately enhancing segmentation performance with limited annotations.

## 6. Conclusions

This study establishes, through comprehensive benchmarking and analysis, that a sequential pretraining strategy—beginning with GPPT on large-scale remote sensing imagery and followed by DAPT on target-domain data—represents the most effective paradigm for semantic segmentation, with its advantages being most pronounced in low-label regimes. Our systematic comparison demonstrates that this GPPT+DAPT framework consistently surpasses both single-phase approaches and the alternative two-phase paradigm initialized from ImageNet. The proposed EGMIM method further augments this framework by improving local feature learning during DAPT, yielding significant gains specifically under extreme label scarcity. Beyond empirical validation, our diagnostic analyses of attention patterns, feature distribution shifts, and parameter evolution offer explanatory insights into why and how this framework works best, revealing that GPPT establishes domain-native spatial priors while DAPT performs efficient, targeted feature-space adaptation.

Crucially, the DAPT phase exhibits high computational efficiency, enabling rapid adaptation to new target tasks with minimal additional training overhead. This combination of superior performance and practical efficiency makes the proposed framework a powerful and readily deployable solution. The strong performance under label scarcity underscores its core utility: providing a robust, high-quality initial model that can serve as a superior starting point for subsequent semi-supervised or weakly supervised learning pipelines, thereby mitigating the foundational bottleneck of error propagation from poor pseudo-labels.

## Figures and Tables

**Table 1:** Composition of the HRGC for GPPT.

Dataset	Resolution (m)	Geographic Coverage	Sample Count
SpaceNet [48]	0.31	Urban imagery from Las Vegas, Paris, Shanghai, Mumbai, San Juan, Rotterdam and Rio de Janeiro	278 k
Inria [49]	0.3	Multi-city coverage across the United States of America	369 k
miniFrance [50]	0.5	Urban and rural areas from multiple French regions	672 k
AIRS *	0.3	Christchurch, New Zealand metropolitan area	264 k
City-OSM [51]	0.1	High-resolution imagery from Berlin, Chicago, Paris, Tokyo, and Zurich	599 k
LandCover.Ai [52]	0.25–0.5	Urban and rural landscapes in Poland	117 k

* The AIRS dataset was resampled from its native resolution of 0.075 m to 0.3 m for consistency.

**Table 2:** Notation and configuration of different pretraining processes.

Pretraining Phase	Dataset	Epochs	GPU Hours *	Notation of Pretraining
GPPT	ImageNet-22K	800	-	${\mathrm{IN}}^{800}$
	HRGC	400	1224	${\mathrm{HRGC}}^{400}$
	Inria	800	484	${\mathrm{Inria}}^{800}$
DAPT	Potsdam	200	20	${\mathrm{Pot}}^{200}$ $\;\mathrm{or}\;{\mathrm{Pot}}_{EGMIM}^{200}$
	Vaihingen	800	10	${\mathrm{Vai}}^{800}$ $\;\mathrm{or}\;{\mathrm{Vai}}_{\mathrm{EGMIM}}^{800}$
	LandCover.Ai	100	19	${\mathrm{LC}}^{100}$ $\;\mathrm{or}\;{\mathrm{LC}}_{EGMIM}^{100}$
	GID	40	19	${\mathrm{GID}}^{20}$ $\;\mathrm{or}\;{\mathrm{GID}}_{EGMIM}^{20}$

* One GPU hour corresponds to one hour of training on a single NVIDIA Tesla V100 GPU.

**Table 3:** Notation and description of different pretraining configurations.

Pretraining Notation	Description
${\mathrm{IN}}^{800}+\mathrm{SKIP}$	Uses an ImageNet-pretrained backbone as the GPPT phase, which is the most commonly adopted initialization strategy in remote sensing. (see Figure 3a)
${\mathrm{HRGC}}^{400}+SKIP$	Employs ${\mathrm{HRGC}}^{400}$ for GPPT and skip DAPT. This represents a typical single-phase pretraining setup often used in RSI pretraining studies. (see Figure 3b)
$\mathrm{SKIP}+{\mathrm{Pot}}^{200}$	Skips GPPT and uses ${\mathrm{Pot}}^{200}$ for DAPT. This denotes another form of single-phase pretraining adopted in RSI-related work. (see Figure 3c)
${\mathrm{IN}}^{800}+{\mathrm{Pot}}^{200}$	Uses an ImageNet-pretrained backbone as the GPPT phase and ${\mathrm{Pot}}^{200}$ for DAPT. This represents the two-phase pretraining setup proposed in several works [7,8,9].
${\mathrm{HRGC}}^{400}+{\mathrm{Pot}}^{200}$	$\mathrm{Applies}\;{\mathrm{HRGC}}^{400}$$\mathrm{for}\;\mathrm{GPPT}\;\mathrm{and}\;{\mathrm{Pot}}^{200}$ for DAPT. This configuration represents our proposed two-phase pretraining framework.

**Table 4:** Mean F1-score (%) on the Potsdam validation set under different labeling ratios. “-”: not conducted.

Pretraining Framework	Ratio of Labels in the Training Set						
	1/100	1/32	1/16	1/8	1/4	1/2	1
${\mathrm{IN}}^{800}+\mathrm{SKIP}$	80.53±0.30	85.22±0.18	86.02±0.16	86.91±0.18	87.68±0.14	88.18±0.07	88.80±0.08
${\mathrm{HRGC}}^{400}+\mathrm{SKIP}$	77.50±0.27	83.39±0.26	85.31±0.18	86.28±0.12	87.02±0.16	87.58±0.08	87.87±0.07
$\mathrm{SKIP}+{\mathrm{Pot}}^{800}$	-	-	-	-	-	-	87.48±0.05
${\mathrm{IN}}^{800}+{\mathrm{Pot}}^{200}$	81.07±0.27	86.15±0.15	87.18±0.16	87.90±0.13	88.79±0.12	89.07±0.09	89.29±0.07
${\mathrm{HRGC}}^{400}+{\mathrm{Pot}}^{200}$ (Ours1)	84.24±0.23	87.89±0.10	88.77±0.08	89.25±0.10	89.93±0.08	90.23±0.06	90.35±0.07
${\mathrm{HRGC}}^{400}+{\mathrm{Pot}}_{EGMIM}^{200}$ (Ours2)	84.85±0.16	88.33±0.11	88.96±0.06	89.44±0.09	89.91±0.05	90.12±0.05	90.31±0.04

**Table 5:** Mean F1-score (%) on the Vaihingen validation set under different labeling ratios.

Pretraining Framework	Ratio of Labels in the Training Set		
	1/4	1/2	1
${\mathrm{HRGC}}^{400}+\mathrm{SKIP}$	80.69±0.13	82.41±0.15	83.30±0.14
${\mathrm{HRGC}}^{400}+{\mathrm{Vai}}^{800}$	83.73±0.13	84.84±0.14	85.66±0.11
${\mathrm{HRGC}}^{400}+{\mathrm{Vai}}_{\mathrm{EGMIM}}^{800}$	84.26±0.12	85.11±0.13	85.52±0.08

**Table 6:** Mean F1-score (%) on the LandCover.Ai validation set under different labeling ratios.

Pretraining Framework	Ratio of Labels in the Training Set			
	1/100	1/16	1/4	1
${\mathrm{HRGC}}^{400}+\mathrm{SKIP}$	80.75±0.14	86.74±0.12	87.71±0.07	88.06±0.08
${\mathrm{HRGC}}^{400}+{\mathrm{LC}}^{100}$	81.25±0.14	87.33±0.10	88.20±0.05	88.67±0.08
${\mathrm{HRGC}}^{400}+{\mathrm{LC}}_{EGMIM}^{100}$	81.66±0.13	87.62±0.10	88.13±0.05	88.46±0.05

**Table 7:** Mean F1-score (%) on the GID validation set under different labeling ratios.

Pretraining Framework	Ratio of Labeled Images in the Training Set			
	1/400	1/200	1/100	1
${\mathrm{HRGC}}^{400}+\mathrm{SKIP}$	84.56±0.20	86.77±0.18	90.18±0.12	94.05±0.09
${\mathrm{HRGC}}^{400}+{\mathrm{GID}}^{20}$	85.48±0.14	87.89±0.14	91.80±0.11	94.70±0.08
${\mathrm{HRGC}}^{400}+{\mathrm{GID}}_{EGMIM}^{20}$	85.88±0.10	88.18±0.10	92.22±0.08	94.79±0.06

**Table 8:** Mean F1-score (%) on Potsdam validation set with different GPPT configurations under varying labeling ratios.

Pretraining Notation	GPPT Configuration		Ratio of Labels in the Training Set						
	Data	Epochs	1/100	1/32	1/16	1/8	1/4	1/2	1
${\mathrm{Inria}}^{800}+{\mathrm{Pot}}^{200}$	Inria	800	83.36±0.24	87.40±0.15	88.27±0.14	88.99±0.11	89.62±0.10	89.82±0.08	89.89±0.08
${\mathrm{HRGC}}^{100}+{\mathrm{Pot}}^{200}$	HRGC	100	83.19±0.24	86.86±0.13	87.85±0.12	88.70±0.12	89.18±0.09	89.50±0.07	89.63±0.07
${\mathrm{HRGC}}^{400}+{\mathrm{Pot}}^{200}$	HRGC	400	84.24±0.23	87.89±0.10	88.77±0.08	89.25±0.10	89.93±0.08	90.23±0.06	90.35±0.07

**Table 9:** Mean F1-score on Potsdam validation set with different EGMIM hyper-parameters under varying labeling ratios. The best performance is highlighted in **bold**.

Hyper-Parameters	Ratio of Labels in the Training Set			
	1/100	1/32	1/16	1/8
w=16 , r=0.05	83.62	87.86	88.74	89.34
w=16 , r=0.10	84.67	87.93	88.81	89.47
w=16 , r=0.15	84.85	**88.33**	**88.96**	**89.44**
w=16 , r=0.20	**85.15**	88.32	88.89	89.44
w=8 , r=0.15	83.04	87.06	88.26	88.76
w=24 , r=0.15	83.86	87.85	88.82	89.33

**Table 10:** Performance comparison of different EGMIM variants (based on ${\mathrm{HRGC}}^{400}+{Pot}_{Algorithm}^{200}$). The best performance is highlighted in **bold**.

Algorithm in DAPT	Ratio of Labels in the Training Set			
	1/100	1/32	1/16	1/8
EGMIMv1	83.90	87.77	88.47	89.18
EGMIMv2	83.88	87.96	88.86	89.40
EGMIM	**84.85**	**88.33**	**88.96**	**89.44**

**Table 11:** Comparison with state-of-the-art methods on the Potsdam dataset under different labeling ratios. Results for our methods (Ours1 and Ours2) are reported as mean ± standard deviation over three runs. Results for other methods are from a single run under our unified evaluation protocol.

Method	Backbone	Segmentation Model	Ratio of Labels in the Training Set					
			1/100		1/16		1	
			mIoU	mF1	mIoU	mF1	mIoU	mF1
TOV [43]	ResNet-50	DeeplabV3+	64.50	77.88	78.97	88.07	79.89	88.65
CMID [20]	ResNet-50	DeeplabV3+	66.85	79.61	78.80	87.98	81.55	89.72
GeRSP [37]	ResNet-50	DeeplabV3+	65.77	78.76	79.28	88.28	81.24	89.51
Cross-Scale MAE [42]	ViT-B	FCN	59.19	73.89	75.54	85.97	77.34	87.10
CSPT [9]	ViT-B	FCN	62.44	76.23	77.94	87.47	78.66	87.92
GFM [40]	swin-B	FCN	72.04	83.58	76.44	86.51	81.40	89.60
SatlasPretrain [60]	swinV2-B	FCN	70.54	82.59	78.83	87.97	80.34	88.96
RingMo [35]	swinV2-B	FCN	63.25	77.41	74.62	85.37	78.56	87.87
CtxMIM [36]	swinV2-B	FCN	65.14	78.77	78.45	87.79	81.68	89.76
Two-phase (Ours1)	swinV2-B	FCN	73.01 ± 0.34	84.24 ± 0.23	80.05 ± 0.13	88.77 ± 0.08	82.62 ± 0.11	90.35 ± 0.07
EGMIM (Ours2)	swinV2-B	FCN	73.95 ± 0.24	84.85 ± 0.16	80.34 ± 0.10	88.96 ± 0.06	82.54 ± 0.05	90.31 ± 0.04

**Table 12:** Comparison of total training time, memory usage, and segmentation performance (mF1) for different pretraining frameworks on the Potsdam dataset (100% labeling ratio). GPPT and DAPT times are in 4-GPU hours; finetuning time is in single-GPU hours. Memory usage is measured per GPU.

Pretraining Framework	GPPT Time	DAPT Time	Finetuning Time	Total Time	Marginal Time	mF1
${\mathrm{IN}}^{800}+\mathrm{SKIP}$	0	0	5	5	5	88.80
${\mathrm{HRGC}}^{400}+\mathrm{SKIP}$	306	0	5	311	5	87.87
$\mathrm{SKIP}+{\mathrm{Pot}}^{800}$	0	18	5	23	23	87.48
${\mathrm{IN}}^{800}+{\mathrm{Pot}}^{200}$	0	5	5	10	10	89.29
${\mathrm{HRGC}}^{400}+{\mathrm{Pot}}^{200}$ (Ours)	306	5	5	316	10	90.35
Memory Usage (per GPU)	24.7 G	24.7 G	7.7 G	-	-	-

## Data Availability

All data used in this study are available from the public repositories. The code presented in this study is available at https://github.com/PZLiuYi/EGMIM/ (accessed on 30 December 2025).

## Abbreviations

The following abbreviations are used in this manuscript:

RSI	Remote sensing image
CNN	Convolutional neural network
VFM	Visual foundation model
FCN	Fully convolutional network
GPPT	General-purpose pretraining
DAPT	Domain-adaptive pretraining
CL	Contrastive learning
MIM	Masked Image Modeling
EGMIM	Edge-Guided Masked Image Modeling
EGM	Edge-Guided Mask
HRGC	High-Resolution Geo-Collection
GID	Gaofen Image Dataset
RPB	Relative position bias
SOTA	State-of-the-art
SSL	Self-Supervised Learning
UDA	Unsupervised Domain Adaptation
